# Differential diagnosis of ameloblastoma and odontogenic keratocyst by machine learning of panoramic radiographs

**DOI:** 10.1007/s11548-021-02309-0

**Published:** 2021-02-06

**Authors:** Zijia Liu, Jiannan Liu, Zijie Zhou, Qiaoyu Zhang, Hao Wu, Guangtao Zhai, Jing Han

**Affiliations:** 1 School of Electronic Information and Electrical Engineering, Shanghai Jiao Tong University, Shanghai, China; 2 Shanghai Ninth People’s Hospital, Shanghai Jiao Tong University School of Medicine, Shanghai, China; 3 College of Stomatology, Weifang Medical University, Weifang, China

**Keywords:** Ameloblastoma, Odontogenic keratocyst, Machine learning, Deep learning

## Abstract

**Purpose:**

The differentiation of the ameloblastoma and odontogenic keratocyst directly affects the formulation of surgical plans, while the results of differential diagnosis by imaging alone are not satisfactory. This paper aimed to propose an algorithm based on convolutional neural networks (CNN) structure to significantly improve the classification accuracy of these two tumors.

**Methods:**

A total of 420 digital panoramic radiographs provided by 401 patients were acquired from the Shanghai Ninth People’s Hospital. Each of them was cropped to a patch as a region of interest by radiologists. Furthermore, inverse logarithm transformation and histogram equalization were employed to increase the contrast of the region of interest (ROI). To alleviate overfitting, random rotation and flip transform as data augmentation algorithms were adopted to the training dataset. We provided a CNN structure based on a transfer learning algorithm, which consists of two branches in parallel. The output of the network is a two-dimensional vector representing the predicted scores of ameloblastoma and odontogenic keratocyst, respectively.

**Results:**

The proposed network achieved an accuracy of 90.36% (AUC = 0.946), while sensitivity and specificity were 92.88% and 87.80%, respectively. Two other networks named VGG-19 and ResNet-50 and a network trained from scratch were also used in the experiment, which achieved accuracy of 80.72%, 78.31%, and 69.88%, respectively.

**Conclusions:**

We proposed an algorithm that significantly improves the differential diagnosis accuracy of ameloblastoma and odontogenic keratocyst and has the utility to provide a reliable recommendation to the oral maxillofacial specialists before surgery.

## Introduction

Ameloblastoma (AB) and odontogenic keratocyst (OK) are both clinically common benign odontogenic lesions [1], which may occur in any part of the jaws, with the highest prevalence in the posterior ramus and body of the mandible. Due to the significant differences in biological behaviors, the two diseases have different treatment strategies. Therefore, how to diagnose and differentiate between the two diseases before surgical intervention is very important. Panoramic radiographs are the most commonly used and convenient imaging examination before surgery. Although there are many discussions on their imaging features in textbooks and in the literatures, it is often difficult to identify them in imaging diagnosis.

In past studies, computed tomography (CT) and magnetic resonance imaging (MRI) were employed to distinguish between these two tumors [2–6]. Some researchers proposed classification methods based on image features. Minami et al. [3] analyzed the MRI imaging of the tumors and summarized the differences between AB, OK, primordial cysts, and radicular cysts on the image. Ariji et al. [6] classify AB and OK by extracting manual features on CT images and then using logistic regression analysis.

With the development of artificial intelligence in recent years, deep learning has shown excellent performance in the fields of medical image classification, segmentation, object detection, etc. [7] Deep learning simulates biological nerves by designing algorithms. It sets multiple hidden layers representing different functions, each of which is composed of many neurons, and each neuron has independent weights. The weights are automatically adjusted by the backpropagation algorithm to complete the learning process. As one of the most effective deep learning structures, convolutional neural networks (CNN) have proven to be a powerful feature extractor that discovers low/mid/high-level features from an enormous number of image datasets. However, for many applications, especially medical, it is not easy to gain massive datasets. The smaller dataset means CNN cannot learn enough classification features causing reduced performance. Transfer learning is a common and effective method to address this issue [8].

Transfer learning is one of the more commonly used artificial intelligence algorithms in recent years. General deep learning methods usually initialize the weight first, such as initializing the weights to a constant or initializing the weights to a Gaussian distribution, which is called training from scratch. Rather than training a CNN from scratch, transfer learning firstly trains a model on a large-scale dataset like ImageNet [9] to learn the classification information and then shares ‘prior knowledge’ obtained from the pre-trained model with another task. Specifically, the ‘prior knowledge’ is the network weights of the pre-trained model, and the new task uses these values as the initial weights. In recent years, many researchers have tried to use transfer learning to classify medical images. Ciompi et al. [10] tackled the problem of automatic classification of pulmonary perifissural nodules by using a pre-trained CNN. Hwang et al. [11] proposed an automatic tuberculosis screening system based on transfer learning, which achieved high screening performance in various performance indicators. Huynh et al. [12] provided a stand-alone classifier without a large dataset to classify digital mammographic cancer tumors. Kooi et al. [13] applied transfer learning for discriminating solitary cysts from soft tissue lesions in mammography. At the same time, machine learning algorithms are also beginning to be applied in oral diseases [14]. Lee JH et al. evaluate the detection and diagnosis of three types of odontogenic cystic lesions—odontogenic keratocysts, dentigerous cysts, and periapical cysts [15]. Ariji Y et al. used DetectNet to detect the lesion location and classify the four types of mandibular radiolucent lesions (ameloblastoma, odontogenic keratocyst, dentigerous cysts, and radicular cysts) at the same time [16]. Watanabe H et al. mainly studied the classification of cyst-like lesions, divided into radicular cysts and other lesions [17]. Yang H et al. classified dentigerous cysts, odontogenic keratocyst, ameloblastoma, and no lesion, using the YOLO network [18]. However, few studies focus on the classification of AB and OK using the deep learning method.

In this paper, novel research based on deep transfer learning was proposed. Considering that CT and MRI are costly and not as convenient as panoramic radiographs, the efficiency will be greatly increased if these two tumors can be distinguished directly on the panoramic radiographs. In this case, panoramic radiographs were employed to this study. Since it is difficult for human eyes to identify AB and OK in panoramic radiographs and there are no enough data, we used transfer learning which can discover high-level features and does not require massive amounts of data. Furthermore, a novel parallel structure based on transfer learning was provided. Two parallel branches use a pre-trained VGG-19 model and a pre-trained ResNet-50 model, which was trained by ImageNet.

## Materials and methods

### Patient cohorts

A total of 420 panoramic radiographs (209 images with AB, and 211 images with OK) from 401 patients were obtained from Shanghai Ninth People’s Hospital. All lesions were located in the mandible and diagnosed between 2012 and 2018. All cases have been confirmed by histopathological analysis, among which AB cases include all subtypes. All examinations were performed on the same panoramic equipment. All images were reviewed by the radiologists, and the images clearly showed the lesion area to be included in the study.

### Data preprocessing and augmentation

**Fig. 1:**
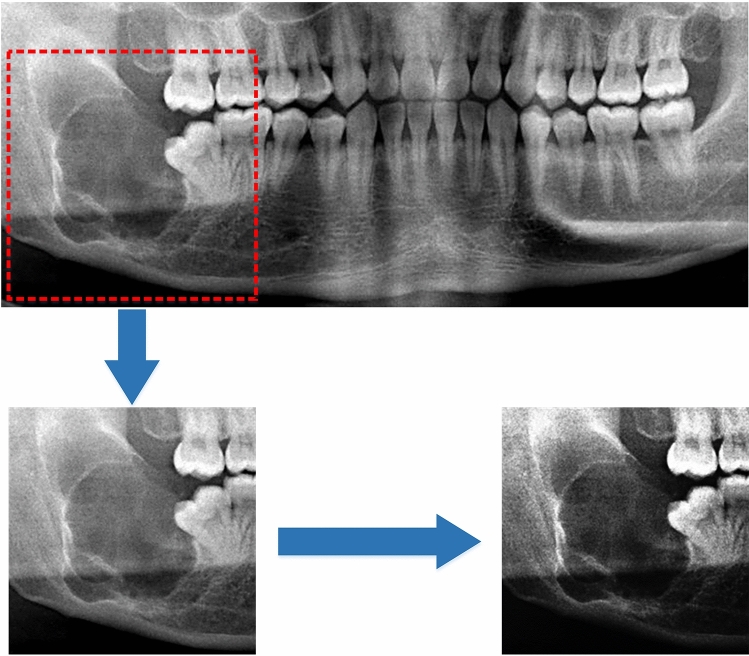
ROI was preprocessed after cropping out from the raw image

The original image has a high resolution, causing the image to occupy a large amount of memory, which will exceed the GPU memory limit. In order to solve the memory problem, there are generally two options. The first one is to resize the image to a smaller size to reduce the memory footprint, which will lead to a reduction in resolution and will inevitably cause loss of picture details. The second option is to crop out the lesion area from the image. In panoramic radiographs, there are many oral tissues such as teeth, gums, and facial bones, etc. Among them, the area of the lesion is relatively small. If only the lesion area is reserved as the research object, it can not only maintain the resolution without losing the details of the tumor texture but also effectively reduce the memory footprint. From this, we defined a region of interest (ROI) performed by expert radiologists. Each image was cropped to a 256 × 256 patch that is centered on the tumor. Furthermore, inverse logarithm transformation and histogram equalization were employed to increase the contrast of the ROI, which can make the texture of the tumor clearer. Figure 1 shows the processing steps.

Generally, CNN needs a large dataset (e.g., millions of samples) to meet the perceived requirement, while small datasets may cause overfitting due to the strong fitting ability of CNN. This means that CNN will lack generalization ability due to over-learning the training dataset, resulting in poor performance on the testing dataset. In order to alleviate overfitting, we applied the data augmentation technique, which aimed to increase the dataset by using geometric transformation to the images in the dataset. The augmentation transformation algorithm usually adopts some simple digital image processing techniques, such as color transformation, and geometric transformation, etc. Considering that image rotation and flipping (horizontal and vertical) will not affect the diagnostic results of the radiologist, we used these two transformations to augment the dataset. During training, the training dataset of every batch was augmented before inputting it into the CNN framework. In other words, each image in this batch of data randomly selected an angle from 0 to 359° to rotate and then choose to flip horizontally or vertically. The transformed images were used only in the current training step and not stored. This real-time augmentation method is different from traditional data augmentation. For example, the traditional data augmentation method usually selects several specific rotation angles (such as 30°, 60°, 90°) and then stores it with the original data. In this way, the data become four times the original data. It can be seen that the transformation of our method is more diverse. In addition, the augmentation strategies were not performed on the validation dataset and testing dataset.

### Transfer learning

Unlike training the network from scratch, transfer learning first trains the parameters of the model on a large-scale dataset to enable the model to obtain the ability to extract features in advance and then uses this model for the target dataset [8]. This way of pre-training a model can effectively solve the problem that the dataset is limited to a small number. Unfortunately, large-scale datasets are dominated by natural images that are significantly different from medical images. Weights transferred from the pre-trained model are optimized to recognize features of the natural images, but lack of the ability to recognize specific classes like images of the maxillofacial tumor. So, it is not appropriate to simply using the weights transferred from the pre-trained model only. To overcome this issue, it is common to freeze weights from connected layers of the pre-trained model ensuring the basic capability of recognition and then add new layers to retrain with backpropagation. In this case, the features unique to the current task images can be learned from the new layer.

**Fig. 2:**
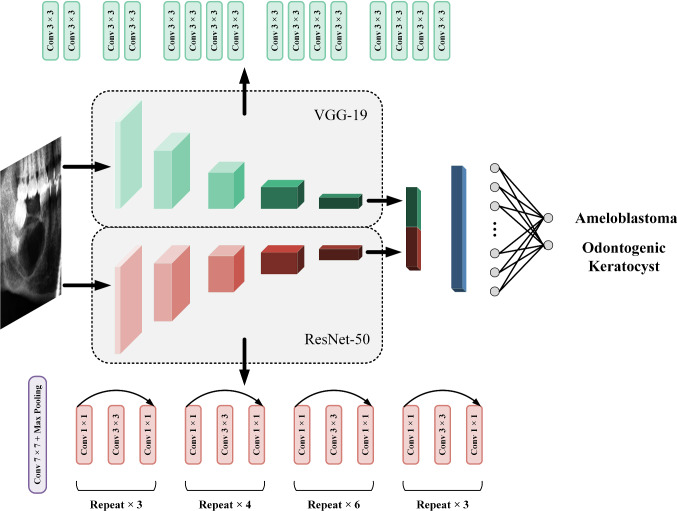
An overview of the proposed network. The parallel structure consists of two branches, VGG-19 and ResNet-50. After these two pre-trained models extract features, they generate feature maps, respectively (red and green rectangles), then concatenate two feature maps together, and connect two convolutional layers (blue rectangles). A global average pooling layer transforms the feature maps into a vector, and the vector finally outputs the prediction score through a fully connected layer. The top of the figure shows the network structure of VGG-19, while the bottom shows the network structure of ResNet-50

Our method is summarized in two steps: one is to design a pre-trained model and the other is to design new layers on top of the pre-trained model. In this study, 19-layer Visual Geometry Group Network (VGG-19) and 50-layer Deep Residual Network (ResNet-50) were applied to be the pre-trained model [19] [20]. As shown in Fig. 2, these two pre-trained models were connected in parallel and extracted features individually. The weights of VGG-19 and ResNet-50 were all pre-trained on the ImageNet database [9]. The structure of the original VGG-19 totally contains five convolutional blocks (first two blocks each have two convolutional layers, and the third to fifth blocks have four convolutional layers, respectively) and three fully connected layers. Each convolutional layer has a 3 × 3 receptive field, and the max-pooling layer of size 2 × 2 is applied behind every convolutional block. ResNet usually performs well in classification tasks, and it solves the degradation problem caused by network deepening through residual connections [20]. The structure of ResNet-50 is illustrated in Fig. 2. After extracting features separately, these two branches were concatenated together. In practice, three fully connected layers of the original VGG-19, average pooling layer and fully connected layer of original ResNet-50 were removed, and the weights of remaining hidden layers from these two pre-trained models were frozen. Freezing weights means that the weights are not updated as the training progresses. On the other hand, two convolutional layers of size 3 × 3, a global average pooling layer, and a fully connected layer with soft-max activation are stacked as new layers connecting the pre-trained model. Specifically, the fully connected layer outputs a two-dimensional vector activated by the soft-max function which corresponds to the prediction score of AB and OK. The sum of these two prediction scores is 1, so 0.5 is selected as the threshold. The class with a prediction score greater than 0.5 is considered the result of model prediction.

### Implementation details

Totally 420 panoramic radiographs were randomly split into training dataset (146 AB and 149 OK), validation dataset (22 AB and 20 OK), and testing dataset (41 AB and 42 OK) in a 7:1:2 ratio. To sum up, the number of images in the training dataset, validation dataset, and testing dataset is 295, 42, and 83, respectively. The original resolution of the images is high and not consistent, about 1500 × 1500. Each of the images requires data preprocessing operations, including ROI extraction, inverse logarithmic transformation, and histogram equalization. After preprocessing, the image resolution before training is 256 × 256. In the training process, the images in the training dataset were input into the network in batches, and the batch size was set to 8. The class of each image was predicted by the above network structure, and the loss value was calculated. The loss value is used to measure the error between the predicted value and the actual value. Then, the network applied the backpropagation algorithm to update the weight value to reduce the loss value, so as to achieve the purpose of optimizing the predicted performance of the network. After all the data in the training dataset have been trained once, it is defined as an epoch. At the end of each epoch, the validation dataset was applied to test the prediction results of the model, including validation accuracy and validation loss value. If the validation loss does not decrease for 20 consecutive epochs, the training stops. The epoch with the lowest loss on the validation dataset was selected to be the final model. All proposed transfer learning algorithms were implemented in Keras framework with Tensorflow backend. Weights of retrained from unfrozen layers were optimized by Adam algorithm, and cross-entropy was adopted to be loss function. The learning rate was set to 0.0001. Rectification nonlinearity (ReLu) activation and batch normalization (BN) were used for all convolutional layers [21, 22]. An Nvidia Titan XP was used to perform the experiments.

## Results

**Fig. 3:**
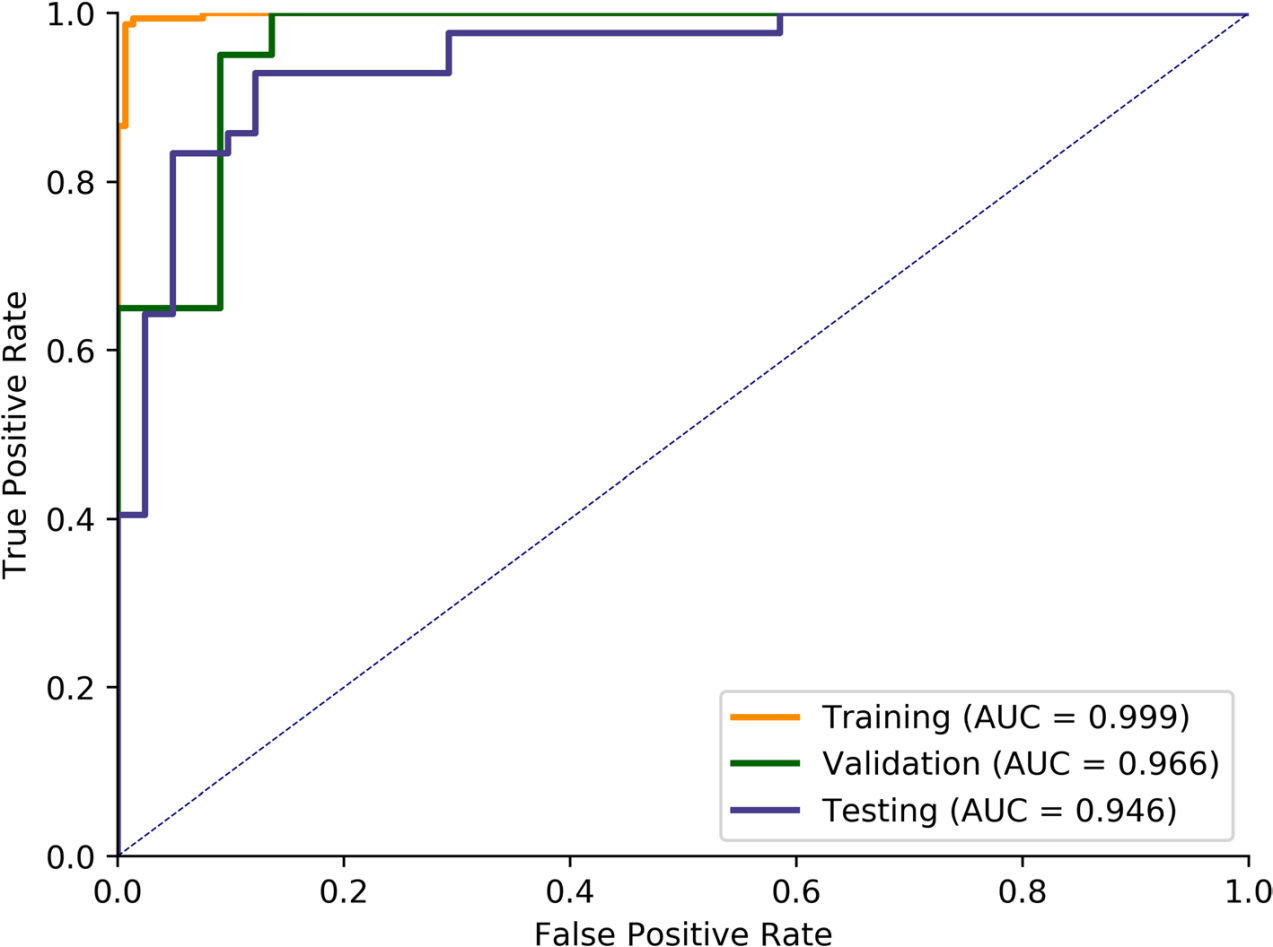
ROC curve and AUC value of training, validation and test set

A parallel network based on transfer learning was proposed to classify AB and OK. Table 1 shows the performance of our model on the training, validation, and test datasets. After 92-epoch training, the proposed network achieved an accuracy of 90.36%, while sensitivity, specificity and F1 score were 92.88%, 87.80%, and 90.70%, respectively. We also plot the receiver operating characteristic (ROC) curve and calculate the area under ROC curve (AUC) value. As shown in Fig. 3, it can be seen that our algorithm achieved an AUC of 0.998, 0.966, and 0.946 on the training set, validation set, and test set, respectively. All these metrics were calculated by Scikit-learn [23]. Furthermore, the average prediction time for a single image is 0.15 s using our classification algorithm.

**Table 1:** Performance on the training, validation, and test datasets

	Accuracy (%)	Sensitivity (%)	Specificity (%)	F1 score (%)
Training set	96.27	99.33	93.15	96.42
Validation set	92.86	95.00	90.91	92.68
Testing set	90.36	92.86	87.80	90.70

**Fig. 4:**
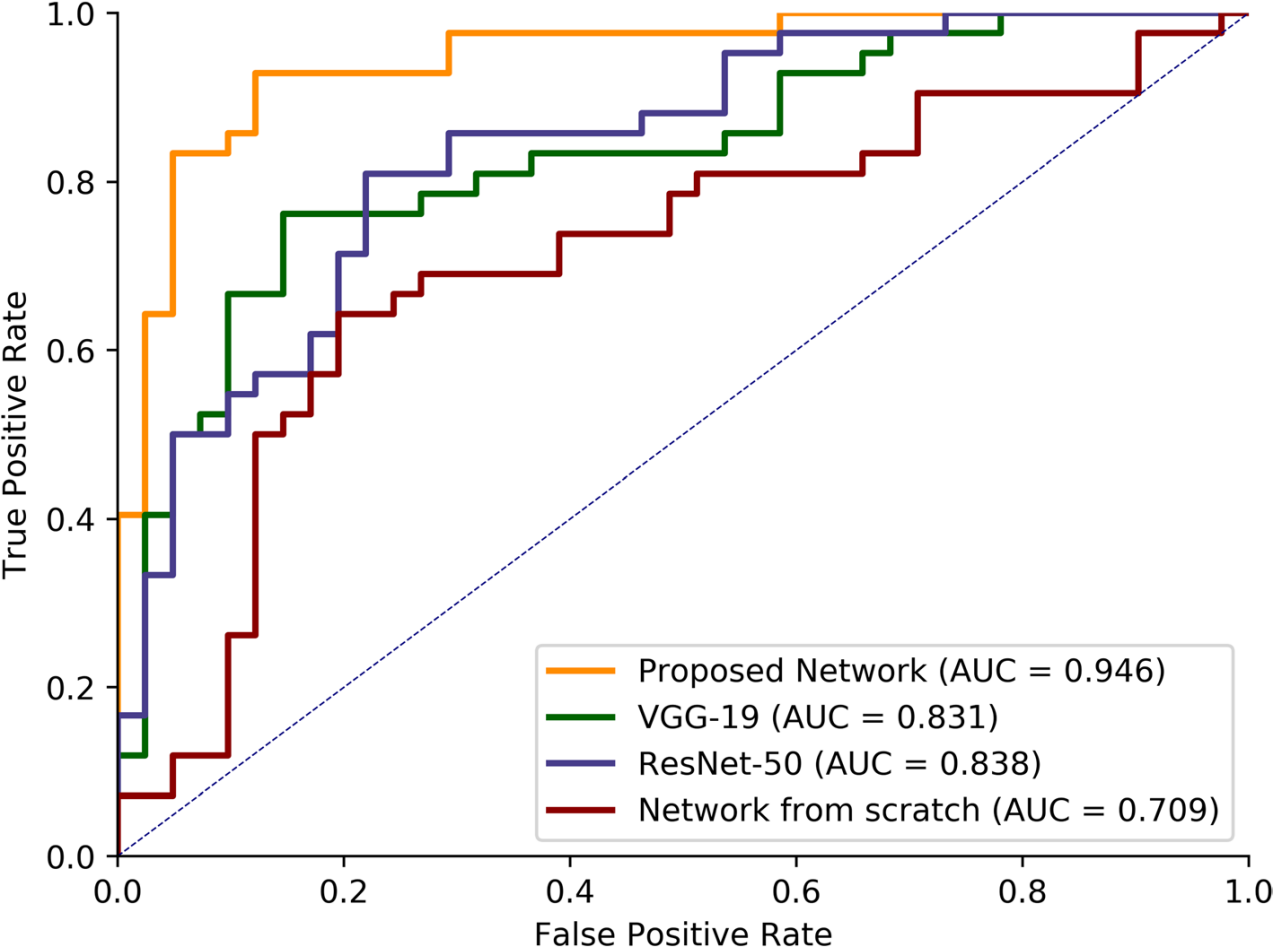
Comparison of ROC curves and AUC value between the proposed network, single pre-trained model and the network trained from scratch

Moreover, to demonstrate that the parallel structure performs better than a single pre-trained model, two branches, VGG-19 and ResNet-50, were trained individually. Each of them uses pre-trained weights from the ImageNet database, the same as our proposed network. As shown in Table 2, the VGG-19 and ResNet-50 give accuracy of 80.72%, 78.31%, respectively, while our model gives an accuracy of 90.36%. As shown in Fig. 4, the AUC of these three networks is 0.946, 0.831 and 0.838, respectively.

**Table 2:** Comparison with two single pre-trained models and the model trained from scratch

	Accuracy (%)	Sensitivity (%)	Specificity (%)	F1 score (%)
VGG-19	80.72	76.19	85.37	80.00
ReNet-50	78.31	85.71	70.73	79.99
Proposed network	90.36	92.86	87.80	90.70
Network from scratch	69.88	66.67	73.17	69.14

In addition, to demonstrate that transfer learning has more advantages than training a model from scratch, a network was built. Instead of initializing with pre-trained weights, this network initialized its weights from a Gaussian distribution and has the same structure as our proposed model. As can be seen from Table 2, compared with the other three transfer learning models, the performance of the network trained from scratch is significantly weaker, with an accuracy of 69.88%.

## Discussion

AB and OK, as two common odontogenic lesions seen in the mandible, have many similarities in clinical manifestations and imaging examinations. AB is the most common odontogenic tumor characterized by expansion and a tendency for local recurrence. OK is an odontogenic cyst representing the third most common cyst of the jaws [1]. Imaging examinations are extremely important for managing intraosseous lesions, with panoramic radiography being used most frequently [24]. AB and OK can both show changes in jaw bone density. In general, AB is manifested as obvious jaw swelling, multilocular lesions and high frequency of root absorption. However, OK mostly grew along the long axis of the jaw bone, with less division and lower rate of root absorption [25].

Surgical management is the only effective method in the treatment for odontogenic tumors, but how to choose an effective surgical method is a problem that clinicians should consider carefully. The treatment plan mainly includes the radical operation of partial resection of the jaw bone and the preservation surgery of decompression combined with curettage. Although AB is a benign tumor, it is locally invasive and has a high recurrence rate after conservative treatment, so partial resection of the jaw bone is often performed [26]. However, more conservative surgical methods were used for OK [27]. Because of the different treatment principles of the two lesions, it is very important to find a more accurate preoperative differential diagnosis method.

Previous researchers have attempted to extract image features to differentiate these two types of tumors. Ariji et al. [6] extracted features such as location, size, number of locules, bone expansion, etc., and then used logistic regression to analyze the features. On the one hand, this method is based only on low-level features that can be observed by the human eye. This way of manually designing features is subjective and cannot describe features in the image that are beyond the cognitive scope of the researchers. On the other hand, logistic regression is a linear method with limited ability to fit complex data. Our method used a convolutional neural network to automatically extract features from images. These features are objective, and the high-level features can be captured due to the depth of the network. At the same time, the ReLu activation layer in the network ensures that the classifier is nonlinear, which enhances the fitting ability of the classifier [18]. With the development of artificial intelligence algorithms, the field of tumor classification and recognition based on digital images has achieved great success. Poedjiastoeti et al. [28] used transfer learning for classifying jaw tumors. Compared with traditional machine learning methods that extract manual features [6], their deep learning algorithms have an overwhelming advantage. In this study, our algorithm obtained a similar result, indicating that the high-level features extracted by CNN can better predict the AB and OK. Compared with their algorithm, we used a deeper (they used the VGG16 structure while the deeper VGG-19 and ResNet-50 structures were applied in our study) and wider (the VGG-19 and ResNet-50 structures in parallel) network structure, which improves the performance of the network.

According to previous research, our experiment chose to use the transfer learning model, which is a kind of CNN structure and a nonlinear structure. Furthermore, considering that AB and OK are relatively similar in panoramic X-ray photographs, we tried to innovate the network structure on the basis of traditional transfer learning and proposed a parallel structure, using two more advanced pre-trained models to extract features simultaneously. From the experiment, our proposed parallel structure proved to be superior to a single pre-trained model. Furthermore, when using the same structure, transfer learning is better than training from scratch. To our knowledge, the accuracy we achieved is higher than all previous studies, which can provide oral maxillofacial specialists with more reliable recommendations before surgical intervention. Considering that some odontogenic and non-odontogenic lesions can present images similar to an AB and OK, in future research, we will expand the number and types of samples and further improve the accuracy of the algorithm.

## Conclusion

In this study, we described a network based on deep learning to automatically classify AB and OK, demonstrating that our algorithm can provide a reliable recommendation to the oral maxillofacial specialists before surgery. In this network, we choose a transfer learning algorithm that performs satisfactorily on a small dataset and simultaneously proposed a parallel structure. VGG and ResNet, as two excellent classification networks, are designed as two parallel branches of our network. Finally, for comparison, we trained two branches of the parallel structure separately and also trained a parallel structure from scratch. It can be observed from the result the proposed network shows excellent performance.
